# Medical Legislation

**Published:** 1848

**Authors:** 


### ARTICLE III.

### MEDICAL LEGISLATION.

The Ohio State Medical Convention at its last session, appointed a committee to prepare and present to the legislature of that state, a memorial soliciting the passage of "a law regulating the practice of physic and surgery in the state of Ohio."

It appears that the committee discharged their duty, and petitions, praying for the same thing, were sent up from various parts of the state. These were, with the memorial of the committee of the convention, referred to the standing committee "on medical societies and medical colleges," which made a report, at some length, arguing the importance of the evils of quackery to the community, and the necessity of their suppression; but come to the conclusion that they are beyond the control of legislation, and that the medical profession must look to itself for a cure of these evils.

They say that there are ignorant, misguided, half-read doctors let loose upon community from the offices of respectable physicians, and, therefore, all laws suppressing quackery would be of no avail.

Now, it would be a nice question to make a law that would prevent unqualified practitioners from exercising the functions of the physician, without including such as these, and it would be strange indeed if our friends of the profession in Ohio would ask a law that would not be operative on such as above referred to.

The proposition that the medical profession must look to itself for a cure of the evils of quackery, is absurd, let it come from never so respectable a source. That it may do much is true, but to ask it, with the means of control it has at command, to suppress the thousand gross impositions continually practiced on community by the nostrum venders, and the host of ignorant pretenders who boast loud because regardless of truth, and who cannot be at once detected by non-professional men, because they have not the knowledge necessary to enable them to judge, is certainly another requirement to "make brick without straw."

The true policy of the physician, certainly is, to explain to his patients, as far as possible, the nature and seat of disease, and the mode of cure he proposes to adopt. This will secure the confidence of patients generally, and will, at the same time, diffuse intelligence amongst them. And it is his duty, when he sees them exposing themselves to danger by employing quacks or quack remedies, to admonish them. But that this shall do away with the necessity for a law suppressing quackery, is just as reasonable as that, because we have a clergy amongst us, whose doctrines and teachings are against all sin and iniquity, we have no necessity for a criminal code. As well might legislators say to the clergy, because your body is not clear of members who commit sin, you must look to yourselves and not to the legislature for the means of suppressing crime. Certainly, the influence of the clergy, in suppressing vice and immorality is great, but not so great as to do way with the necessity of criminal legislation. And if, in a matter that all profess to understand, there is a universally ackowledged necessity for protecting community from being imposed upon, how much plainer the necessity, where the mass of the people cannot be expected to be sufficently informed to judge for themselves.

The question of medical laws is too often regarded in the light of protection to the profession, instead of to the community at large. Physicians have little, if any, personal interest in the passage of laws regulating practice; but, certainly, the whole community is interested in being protected against the depredations of the hoard of ignorant pretenders, by whom lives and limbs are so continually being sacrificed.

We see by an article from the pen of the able Dr. Dawson, of Ohio, in the Western Lancet, that the profession is encouraged to rally for another effort. In this matter duty calls and we hope our friends will respond.

We observe by the newspapers that a physician must, according to a recent decision in one of the New York courts, continue to attend his patient, when once employed, unless the patient refuses to take his presriptions, or the physician gives due notice of his intention to abandon the case. What would be due notice, is, in this case, an important question.

We find, by the journal of the last legislature of Indiana, that a member in the House of Representatives, from one of the interior counties, offered the following resolution :

"*Resolved*, That the committee on benevolent and scientific institutions be instructed to inquire into the expediency of requiring the physicians of this State to attend, without charge, on all persons of their vicinity who are, or may be, sick, and who are not worth more than twenty dollars, exclusive of the necessary wearing apparel of such person and his or her family, and making it an indictable offence for any physician to refuse without a good and sufficient reason therefor."

Which was at once laid on the table.

The same member, not satisfied with this failure to have his proposition simply for instruction entertained, immediately offered the following:

"*Resolved*, That the judiciary committee be instructed to report a bill to this House as soon as may be practicable, reducing the usual charges of physicians as such at least thirty-three per cent."

These efforts of the Honorable Gentleman excited some sport, we understand, but, to the credit of the House, received no further consideration.

Absurd as the propositions in these resolutions are, there are those in the State, as may be inferred by the efforts of this kind made each winter, who seriously think something ought to be done to make the *Doctors* work for nothing in a considerable part of their practice, and for thirty-three per centum less than their services are worth in the remainder. That this should be the case with ignorant and penurious men — those (samples of whom are to be found in almost every community) who are too stingy not only to provide for themselves the ordinary comforts and necessaries of life when in health, but to employ a physician when sick—is not strange; but that a man who can attain to a sufficient standing in society to be elected to the legislature, should publicly propose such a thing, is remarkable indeed.

Let medical men unite against every infringement upon the rights and privileges of the profession, and their duties and obligations to community, whether by quacks or demagogues, and with their intelligence, standing, and extensive acquaintance, they may make their influence felt in such way as to be appreciated. E.

